# *Mameliella sediminis* sp. nov., a novel polyhydroxyalkanoate-accumulating bacterium

**DOI:** 10.1099/ijsem.0.005274

**Published:** 2022-03-09

**Authors:** Wei-shuang Zheng, Sheng-qiang Zhai, Meng-jun Zhang, Yi Huang

**Affiliations:** ^1^​ Marine Institute for Bioresources and Environment, Peking University Shenzhen Institute, Shenzhen 518057, PR China; ^2^​ PKU-HKUST Shenzhen-Hong Kong Institution, Shenzhen 518057, PR China; ^3^​ Tsinghua Shenzhen International Graduate School, Shenzhen 518055, PR China; ^4^​ College of Environmental Sciences and Engineering, Peking University, Beijing 100871, PR China

**Keywords:** *Mameliella*, draft genome sequencing, ANI, dDDH, polyhydroxyalkanoate

## Abstract

A Gram-stain-negative, strictly aerobic, non-motile, rod-shaped bacterium, capable of producing poly-β-hydroxyalkanoate, designated DP3N28-2^T^, was isolated from the sediment collected from Daya Bay, Guangdong, PR China. Optimal growth occurred at 37–40 °C, pH 6.0 and in the presence of 4 % NaCl. The 16S rRNA gene sequences analysis revealed that DP3N28-2^T^ showed highest similarities with *Mameliella alba* DSM 23384^T^ (98.3 %), *Antarctobacter jejuensis* 13–2-B6^T^ (97.2 %), *Antarctobacter heliothermu* s El-219^T^ (96.8 %), *Maliponia aquimaris* MM-10^T^ (96.7 %), *Ponticoccus litoralis* CL-GR66^T^ (96.4 %) and *Aquicoccus porphyridii* L1 8-17^T^ (96.1 %). The predominant fatty acids (>10 %) were summed feature 8 (C_18 : 1_ω6*c* and/or C_18 : 1_ω7*c*; 72.1 %) and C_16 : 0_ (11.0 %). The polar lipids contain phosphatidylethanolamine, phosphatidylmonomethylethanolamine, phosphatidylglycerol, one aminophosphlipid, one phospholipid and three unidentified lipids. The respiratory quinone was Q-10. The DNA G+C content was 63.0 mol% (data from the genome sequence). The estimated genome size was 5.12 Mb. The average nucleotide identity values between the DP3N28-2^T^ genome and the genome of *M. alba* was 81.1 %, while the digital DNA–DNA hybridization value was 23.4 %. The phenotypic, genotypic and chemotaxonomic differences between DP3N28-2^T^ and its phylogenetic relatives indicates that DP3N28-2^T^ should be regarded as representing a novel species of the genus *Mameliella *, for which the name *Mameliella sediminis* sp. nov. is proposed. The type strain is DP3N28-2^T^ (=MCCC 1K06218^T^=KCTC 82804^T^).

The genus *Mameliella* was proposed by Zheng *et al*. in 2010, with *Mameliella alba* as the type species [1]. In 2021, the ‘Roseobacter clade’ of the family *Rhodobacteraceae* was validly published as the family *Roseobacteraceae* [2, 3], therefore the genus *Mameliella* was moved to the family *Roseobacteraceae* of the class *Alphaproteobacteria *. In 2015, four heterotypic synonyms of *Mameliella alba *, *Mameliella phaeodactyli* [4], *Mameliella atlantica* [5], *Ponticoccus lacteus* [6] and *Alkalimicrobium pacificum* [7], have been published independently, because the chimaeric 16S rRNA gene sequence of the type strain JLT354-W^T^ (EU734592.1) had been submitted to the GenBank database [8]. After identification of this mistake, by January 2022, the genus *Mameliella* contains only one species with a correct validly published name according to List of Prokaryotic names with Standing in Nomenclature (https://lpsn.dsmz.de/genus/mameliella).

During a course of study on the poly-β-hydroxyalkanoate-producing marine bacteria from Daya Bay, Guangdong, PR China, we isolated a novel bacterial strain resembling members of the genus *Mameliella *, designated DP3N28-2^T^. Using PCR with specific primers [9], we identified that DP3N28-2^T^ had a poly-β-hydroxyalkanoate (PHA) polymerase (*phaC*) gene (gel profile data not shown), which indicated the potential for producing PHA. Therefore, in this study, we were aimed to determine the exact taxonomic position of DP3N28-2^T^, by using a polyphasic approach based on phenotypic, chemotaxonomic and genotypic data, and estimated the PHA-producing ability of the genus *Mameliella *.

## Isolation and enrichment

The novel isolate of a member of the genus *Mameliella* was isolated from a surface sediment sample near off-shore sewage outfall (22°34′19.57″ N, 114°30′29.33″ E) from 5.3 m depth. The sediment sample was incubated in medium containing 5 g (w/w) peptone and 1 g (w/w) yeast extract per litre sea water, at 30 °C for 28 days, and then isolated using the standard dilution plating technique on marine agar (MA; Haibo). After purification by re-streaking, the novel strain was routinely cultivated at 30 °C on MA and stored at −80 °C in sterile 15 % (v/v) glycerol supplemented with 1 % (w/v) saline. The reference type strain, *M. alba* CGMCC 1.7209^T^, were procured from the China General Microbiological Culture Collection Centre. Both strains could form single colonies on MA within 24 h at 30 °C. To standardize the culture conditions in this study, we cultivated *M. alba* CGMCC 1.7209^T^ using MA or MB.

## Phylogeny based on the 16S rRNA gene and genome sequences

**Fig. 1.**
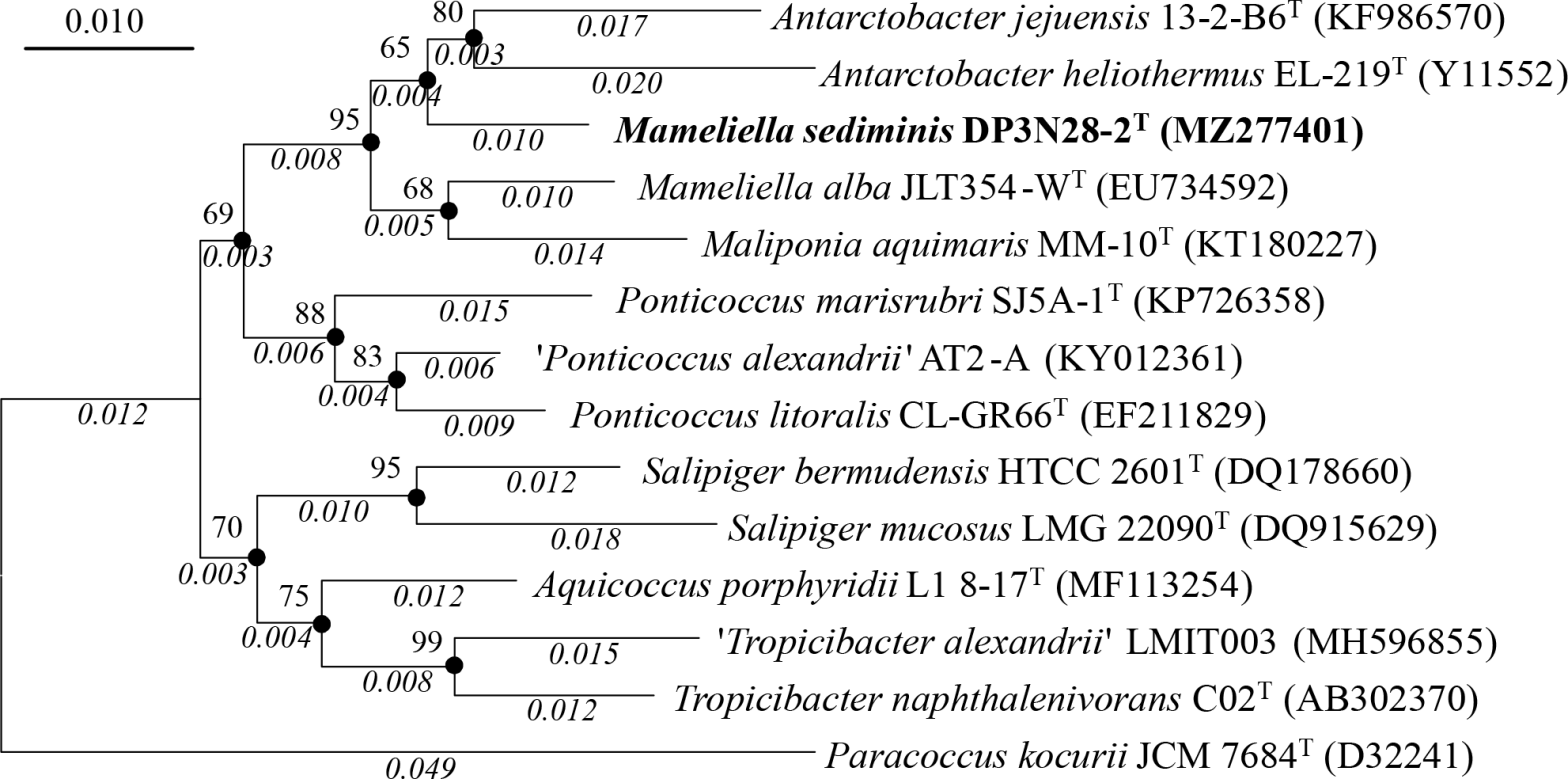
Neighbor-joining phylogenetic tree based on 16S rRNA gene sequences showing the relationships of DP3N28-2^T^ and related species of the family *Roseobacteraceae *. Bootstrap values based on 1000 replicates are listed as percentages at branching points; only bootstrap values above 50 % are shown. All positions containing gaps and missing data were eliminated. There were a total of 1282 positions in the final dataset. Filled circles indicate that the corresponding nodes were also recovered in both maximum-likelihood and maximum-parsimony trees. Values in italics indicate branch length. *Paracoccus kocurii* JCM 7684^T^ (D32241) was used as an outgroup. Bar, 0.01 substitutions per nucleotide position.

**Fig. 2.**
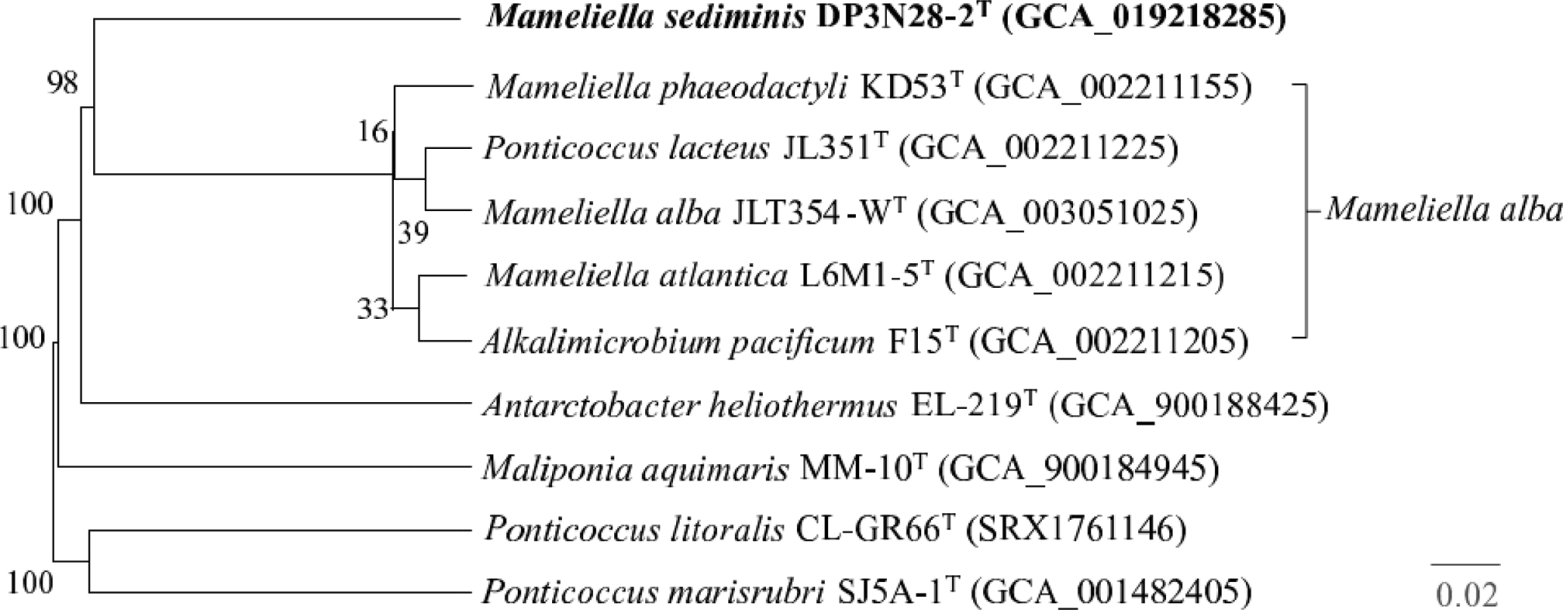
Genome-based phylogenetic tree of DP3N28-2^T^ and related type strains using data from the Type Strain Genome Server. Tree inferred with FastME 2.1.4 from Genome blast Distance Phylogeny (GBDP) distances calculated from genome sequences. Branch lengths are scaled in terms of GBDP distance formula d_5_; numbers above branches are GBDP pseudo-bootstrap support values from 100 replications. GenBank accession numbers are shown in parentheses. Bar, 0.02 substitutions per nucleotide position.

The 16S rRNA gene of DP3N28-2^T^ was amplified by PCR with the universal primers 27F and 1492R [10], and sequenced by Sangon (Shanghai, PR China). The 16S rRNA gene sequences of DP3N28-2^T^ was analysed by using the blast programme (NCBI) and compared with closely related sequences of reference organisms using the service from EzBioCloud (www.ezbiocloud. net) [11]. Alignments of 16S rRNA gene sequences were performed using the programme clustal_x, version 1.81 [12], and positions with insertions or deletions were excluded during calculations. Phylogenetic trees were reconstructed by the neighbor-joining (NJ) [13], maximum-parsimony (MP) [14] and maximum-likelihood (ML) [15] methods with the mega 7 programme package [16]. The NJ tree and ML tree were reconstructed using evolutionary distances that were calculated with the Kimura two-parameter model with Gamma distribution [17]. The MP tree was obtained using the subtree-pruning–regrafting algorithm [14]. All positions containing gaps and missing data were eliminated. The stability of the clusters was evaluated by bootstrap analysis based on 1000 replicates. A nearly complete 16S rRNA gene sequence of DP3N28-2^T^ obtained by amplification (1303 bp) was included in the 16S rRNA gene sequence assembled from genomic sequences (1437 bp). On the basis of the 16S rRNA gene sequence (from genome sequence), strain DP3N28-2^T^ was closely related to *Mameliella alba* DSM 23384^T^ (98.3%), *Antarctobacter jejuensis* 13–2-B6^T^ (97.2%), *Antarctobacter heliothermu* s El-219^T^ (96.8%), *Maliponia aquimaris* MM-10^T^ (96.7%), *Ponticoccus litoralis* CL-GR66^T^ (96.4%) and *Aquicoccus porphyridii* L1 8-17^T^ (96.1%). In the NJ, MP and ML phylogenetic trees (Fig. 1), although the distance between DP3N28-2^T^ and *M. alba* was the shortest, they did not cluster together. Therefore, a genome-based phylogenetic tree was reconstructed using the Type (Strain) Genome Server provided by DSMZ [18] to further verify the phylogenetic position. This tree indicated that DP3N28-2^T^ formed a clade with the type strain of *M. alba* (Fig. 2), which indicated that DP3N28-2^T^ could represent a novel species of the genus *Mameliella *.

The draft genome of DP3N28-2^T^ was sequenced using a HiSeq X-ten platform (Illumina), assembled at Guangdong Magigene Biotechnology. (Guangdong, PR China; http://www.magigene.com/) using the SPAdes software (v3.13.0) [19] and annotated using the NCBI Prokaryotic Genome Annotation Pipeline [20]. The assembled genome of DP3N28-2^T^ (JAHUZG000000000) had a total length of 5 120 735 bp, coverage of 600×, contig N50 value of 1 507 360 bp, 4966 predicted gene sequences and 54 RNAs. The final assembly contained 21 contigs and a had a DNA G+C content of 63.0 mol%.

Average nucleotide identity (ANI) using the OrthoANI algorithm [21] and digital DNA–DNA hybridization (dDDH) values [22] was obtained to determine the genetic relatedness between DP3N28-2^T^ and the type species *M. alba* CGMCC 1.7290^T^ (FMZI01000000). ANI and dDDH (formula 2) were 81.1 and 23.4 %, respectively. They were both lower than the threshold value for species demarcation (ANI, 95–96 %; dDDH, 70 %). Furthermore, the difference in DNA G+C content between DP3N28-2^T^ and the type species was 2.2 mol%, which indicated that they represented two different species [23]. Therefore, on the basis of the results of the phylogenetic analysis, the 16S rRNA gene sequence similarity values and the genomic sequence similarity values, DP3N28-2^T^ was considered to represent a novel species of the genus *Mameliella *.

## Phenotypic and chemotaxonomic characterization

All morphological and physiological features of DP3N28-2^T^ were examined after incubation in MB or on MA at 30 °C for 24 h to a maximum of 1 week unless otherwise specified. Cell morphology, Gram-staining and motility were determined by microscopy (CX43, Olympus) and transmission electron microscopy (Tecnai G2 F30, FEI). Cells of DP3N28-2^T^ were Gram-stain-negative non-flagellated and rod shaped. The cell length was 1.3–4.7 µm and the width was 0.5–0.9 µm (Fig. S1, available in the online version of this article). The shape varied due to the distribution of the PHA granules. Growth of DP3N28-2^T^ and *M. alba* CGMCC 1.7290^T^ was tested at 4, 8, 15, 20, 25, 30, 35, 37, 40, 45 and 50 °C, NaCl concentrations in the range of 0–10 % (w/v; at increments of 1 %) and pH values in the range of 4.0–12.0 (at intervals of 1.0 pH unit) adjusted using HCl or NaOH. The growth was measured using OD_600_ after 24, 48 and 96 h. Growth occurred at temperatures from 15 to 45 °C (optimally at 37–40 °C), pH from 5.0 to 8.0 (optimally at pH 6.0) and NaCl concentrations from 0–10 % (optimally at 4 %). No growth observed after incubation in an anaerobic chamber on MA for 2 weeks. Oxidase activity was determined using a bioMérieux oxidase reagent kit according to the manufacturer’s instructions. Catalase activity was tested by bubble production using 3 % (v/v) H_2_O_2_ solution. Utilization of citrate, pyruvate and malonate as sole carbon sources was determined. DP3N28-2^T^ was cultivated in the minimal salt medium containing, per litre of distilled water: 2.28 g K_2_HPO_4_·3H_2_O, 1.32 g (NH_4_)_2_SO_4_, 0.47 g NaHPO_4_, supplemented with 1 % carbon source. Hydrolysis of agar and starch, H_2_S production, reduction of nitrate, Voges–Proskauer test, methyl red test and indole production were investigated according to the methods described by Dong and Cai [24]. The phenotypic characterization was carried out using API 20NE, API ZYM (bioMérieux) and GEN III MicroPlate (Biolog) on DP3N28-2^T^ and the reference type strain independently twice without replicates at 30 °C. The polyhydroxyalkanoates production of DP3N28-2^T^ was examined as described by Juengert *et al.* [25] using gas chromatography (GC-2014, Shimadzu) with GC column DB-WAX (G6501-CTC, Agilent Technologies).

DP3N28-2^T^ exhibited phenotypic similarities to *M. alba* CGMCC 1.7290^T^, including being Gram-stain-negative, rod shaped, aerobic, having positive reactions for catalase and oxidase and identical reactions in API ZYM tests, except for α-galactosidase. Both strains could accumulate poly-β-hydroxybutyrate. The GC result indicated that DP3N28-2^T^ and *M. alba* CGMCC 1.7290^T^ accumulated PHA up to 12.3±2.9 % and 19.2±5.8 % (per dry cell weight, ±standard deviation), respectively. In the genomic data, the PHA synthase gene (*phaC*), PHA depolymerase gene (*phaZ*), regulator gene (*phaR*) and PHA granule-associated phasin gene (*phaP*) were annotated (accession numbers are listed in Table S1).

On the basis of the data obtained during this study, we could distinguish DP3N28-2^T^ from *M. alba* in various ways (Table 1). However, the reported phenotypic features of *M. alba* were highly variable among the five representatives [8], for example the optimal NaCl (w/v, %) of *M. alba* JLT354-W^T^ was 1–3 [1] while that of *A. pacificum* F15^T^ was 3.5 [7], from which we could draw different conclusion about the similarity between strain DP3N28-2^T^ and *M. alba *. Therefore, we identified three features including being slightly curved, negative for nitrate reduction and preferring acidic growth conditions (pH 6.0) as the critical phenotypic differences between DP3N28-2^T^ and *M. alba *. The detailed physiological and biochemical characteristics of DP3N28-2^T^ are given in Tables 1 and 2, Table S2 and the species descriptions. According to the Biolog GEN III MicroPlate test, DP3N28-2^T^ was susceptible to troleandomycin, nalidixic acid, aztreonam and niaproof 4, and resistant to lincomycin, vancomycin, fusidic acid, rifamycin SV, guanidine HCl and minocycline. Cultures of DP3N28-2^T^ and *M. alba* CGMCC 1.7290^T^ were grown in MB at 30 °C for 48 h, and then freeze-dried for polar lipid, respiratory quinone and cellular fatty acid composition analysis. The cellular fatty acid composition was measured by the Marine Culture Collection of China (MCCC; Xiamen, Fujian, China) using the Microbial ID system [26]. Respiratory quinones of DP3N28-2^T^ was determined by the MCCC using ultra-performance liquid chromatography–tandem mass spectrometry methods [27]. The cellular polar lipids were extracted and determined by two-dimensional thin-layer chromatography using silica gel plates (60 F254, Merck) [28, 29]. Total lipid material was detected using molybdatophosphoric acid, and specific functional groups were detected using ninhydrin and phosphomolybdenum blue reagents [29, 30]. The respiratory quinone of both strains was Q-10. As shown in Table 2, the two strains showed similar cellular fatty acid profiles, with summed feature 8, C_16 : 0_ and C_18 : 0_ as the top three components (>5 %), however, in *M. alba* CGMCC 1.7290^T^, the proportion of C_18 : 0_ was higher than that of C_16 : 0_, which were consistently shown in all the five representatives of *M. alba* [8]. As shown in Fig. S2, the polar lipids of DP3N28-2^T^ included phosphatidylethanolamine, phosphatidylmonomethylethanolamine, phosphatidylglycerol, one aminophospholipid, one phospholipid and three unidentified lipids. The polar lipid composition was variable among the *M. alba* strains [8]*,* but none of them contained phosphatidylmonomethylethanolamine, which could be used to distinguish DP3N28-2^T^ from *M. alba.*

**Table 1.** Characteristics of DP3N28-2^T^ and the type species of the genus *Mameliella.* Strain: 1, *Mameliella* sp. DP3N28−2T; 2, *M. alba* CGMCC 1.7290T. All Data were obtained from this study unless otherwise indicated. +, Positive or grow; −, negative or not grow; W, weakly positive or grow. The detailed data for API ZYM, API 20NE and Biolog GEN III are given in Table S2.

Characteristic	1	2
Cell morphology	Straight to curved rods	Rods
Cell size (μm×μm)	0.5–0.9×1.3–4.7	0.7–0.8×1.0–1.9
Colony colour	Beige	Yellowish white
Motility	−	−
Flagella	−	−
Growth range (optimum)		
Temperature (°C)	37–40	37
pH	6.0	8.0
NaCl (w/v, %)	4	1–3
Nitrate reduction	−	+
Enzymic activities (API ZYM)		
α-galactosidase	+	−
API 20NE test		
d-Glucose	w	+
l-Arabinose	w	+
d-Mannitol	−	+
Maltose	−	+
Potassium gluconate	−	+
Adipic acid	−	w/+*
Malic acid	−	w/+*
Trisodium citrate	−	w/+*
Phenylacetic acid	−	+
DNA G+C content (mol%)	63.0	65.2

*Data after solidus is from reference [8].

**Table 2.** Cellular fatty acid contents (percentages) of DP3N28-2^T^ and *Mameliella alba
* Strains: 1, DP3N28-2^T^; 2, *M. alba* CGMCC 1.7290^T^. All data were obtained during this study. Major fatty acids (>10%) are highlighted in bold type. Only fatty acids exceeding 1.0 % of the total cellular fatty acids of at least one of the strains are shown. tr, Trace (<1.0%); nd, not detected.

Fatty acid	1	2
C_12 : 0_ 3-OH	1.2	nd
C_16 : 0_	**11.0**	8.4
C_18 : 0_	8.5	**13.2**
C_18 : 1_ω7*c* 11-methyl	3.9	2.9
C_19 : 0_cyclo ω8*c*	tr	1.1
Summed feature 8*	**72.1**	**67.5**

*Summed feature 8 contained C_18 : 1_ ω7*c* and/or ω6*c*.

On the basis of these phenotypic, chemotaxonomic, phylogenetic and genetic data, we propose to classify strain DP3N28-2^T^ as representing a novel species of the genus *Mameliella *, for which the name *Mameliella sediminis* sp. nov. is proposed.

## Description of *Mameliella sediminis* sp. nov.

*Mameliella sediminis* (se.di’mi.nis L. gen. neut. n. *sediminis*, of sediment).

Cells are Gram-stain-negative, aerobic, and non-motile rods (0.5–0.9 µm wide and 1.3–4.7 µm long). Small colonies (1–2 mm) are produced on MA after incubation at 30 °C for 24 h. Colonies are smooth, convex and beige. Growth occurs at 15–45 °C, at pH 5.0–8.0 and in the presence of 0–10 % NaCl; optimal growth occurs at 37–40 °C, at pH 6.0 and in the presence of 4.0 % NaCl. Positive for catalase and oxidase. Negative for nitrate reduction, hydrolysis of gelatin and starch, methyl red test, Voges–Proskauer reaction, and H_2_S production. Pyruvate are used as sole carbon source, but not citrate. In the API ZYM tests, positive for alkaline phosphatase, esterase (C4), esterase lipase (C8), lipase (C14), leucine arylamidase, valine arylamidase, cystine arylamidase, acid phosphatase, naphthol-AS-BI-phosphohydrolase and α-galactosidase; negative for trypsin, α-chymotrypsin, β-galactosidase, β-glucuronidase, α-glucosidase, β-glucosidase, *N*-acetyl-β-glucosaminidase, α-mannosidase and α-fucosidase. In the API 20NE tests, positive for d-glucose fermentation, urease, β-glucosidase (aesculin hydrolysis) and β*-*galactosidase; weakly positive for gelatin hydrolysis, utilization of d-glucose, l-arabinose, and d-mannose; negative for reduction of nitrate to nitrite, denitrification, indole production, arginine dihydrolase, utilization of d-mannitol, *N*-acetyl-glucosamine, maltose, potassium gluconate, capric acid, malic acid, adipic acid, phenylacetic acid and trisodium citrate. According to Biolog GEN III MicroPlate assays, α-hydroxybutyric acid, l-arginine, l-lactic acid, β-hydroxy-d,l-butyric acid, d-fucose, acetoacetic acid and trehalose are oxidized. The polar lipids are phosphatidylethanolamine, phosphatidylmonomethylethanolamine, phosphatidylglycerol, one aminophosphlipid, one phospholipid and three unidentified lipids. The main fatty acids are summed feature 8 (C_18 : 1_ω6*c* and/or C_18 : 1_ω7*c*) and C_16 : 0_. The respiratory quinone is Q-10.

The type strain, DP3N28-2^T^ (=MCCC 1K06218^T^=KCTC 82804^T^) was isolated from the sediment collected from Daya Bay, Guangdong, PR China. The DNA G+C content of the type strain is 63.0 mol % (data from genome sequence). The GenBank accession number for the 16S rRNA gene sequence and draft genome of strain DP3N28-2^T^ are MZ277401 and JAHUZG000000000 respectively.

## Supplementary Data

Supplementary material 1
