# Supplementary material for: Mameliella sediminis sp. nov., a novel polyhydroxyalkanoate-accumulating bacterium
Source: Int J Syst Evol Microbiol. 2022 Mar 9;72(3):005274. doi: 10.1099/ijsem.0.005274 (PMC9558576; doi:10.1099/ijsem.0.005274)
Supplement: Supplementary material 1 [file ijsem-72-5274-s001.pdf]

International Journal of Systematic and Evolutionary Microbiology Supplementary Material

***Mameliella sediminis* sp. nov., a novel polyhydroxyalkanoate-accumulating bacterium**

Wei-shuang Zheng[1,2,†], Sheng-qiang Zhai[1,3,†], Meng-jun Zhang[4] and Yi Huang[1,4,*]

[1] Marine Institute for Bioresources and Environment, Peking University Shenzhen Institute, Shenzhen, 518057, PR China

[2] PKU-HKUST Shenzhen-Hong Kong Institution, Shenzhen, 518057, PR China

[3] Tsinghua Shenzhen International Graduate School, Shenzhen, 518055, PR China

[4] College of Environmental Sciences and Engineering, Peking University, Beijing 100871, PR China

Author for correspondence: Yi Huang; Email: yhuang@pku.edu.cn

[†] These authors contributed equally to this work.

Supplementary Fig. S1. Scanning electron micrographs of strain DP3N28-2^T grown on marine agar at 30 °C under aerobic condition for 2 days.

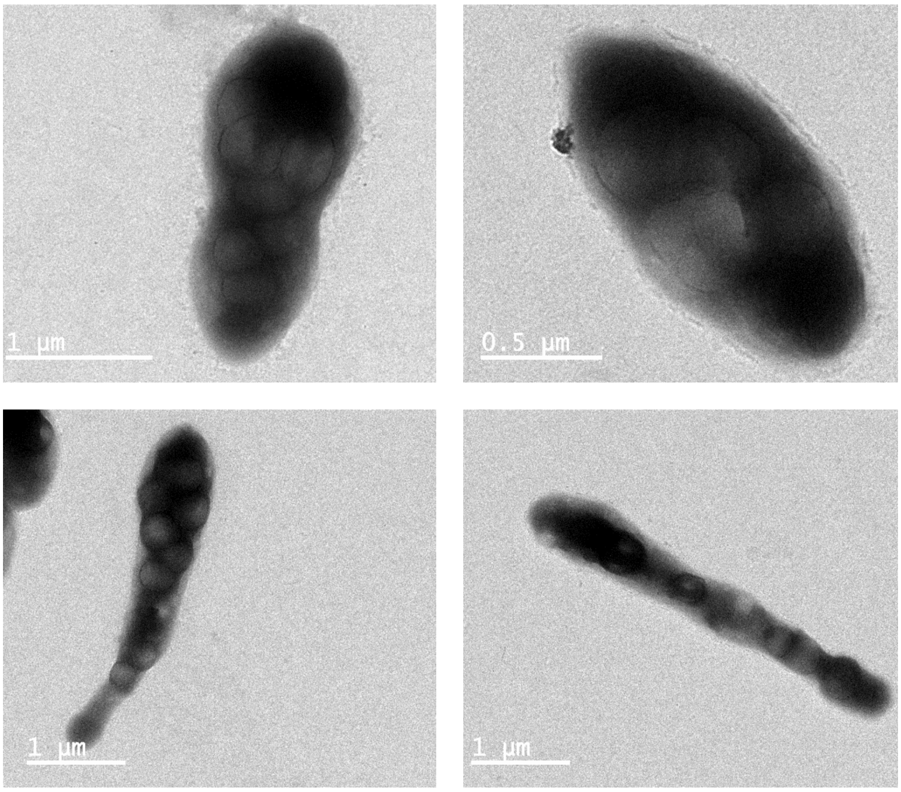

Supplementary Fig. S2. Two-dimensional thin-layer chromatography plate images of lipids: total polar lipids (a), aminolipids (b), phospholipids (c) of *Mameliella* sp. strain DP3N28-2^{T} and total polar lipids (d), aminolipids (e), phospholipids (f) of *M. alba* CGMCC 1.7290^{T}. Ascending solvent system: (I) chloroform/ methanol/water (65:25:4, by vol.); (II) chloroform/methanol/acetic acid/water (85:12:15:4, by vol.). AL, Aminolipid; APL, Aminophospholipid; L, unidentified lipid; PE, phosphatidylethanolamine; PG, Phosphatidylglycerol; PME, Phosphatidylmonomethylethanolamine; PL, phospholipid.

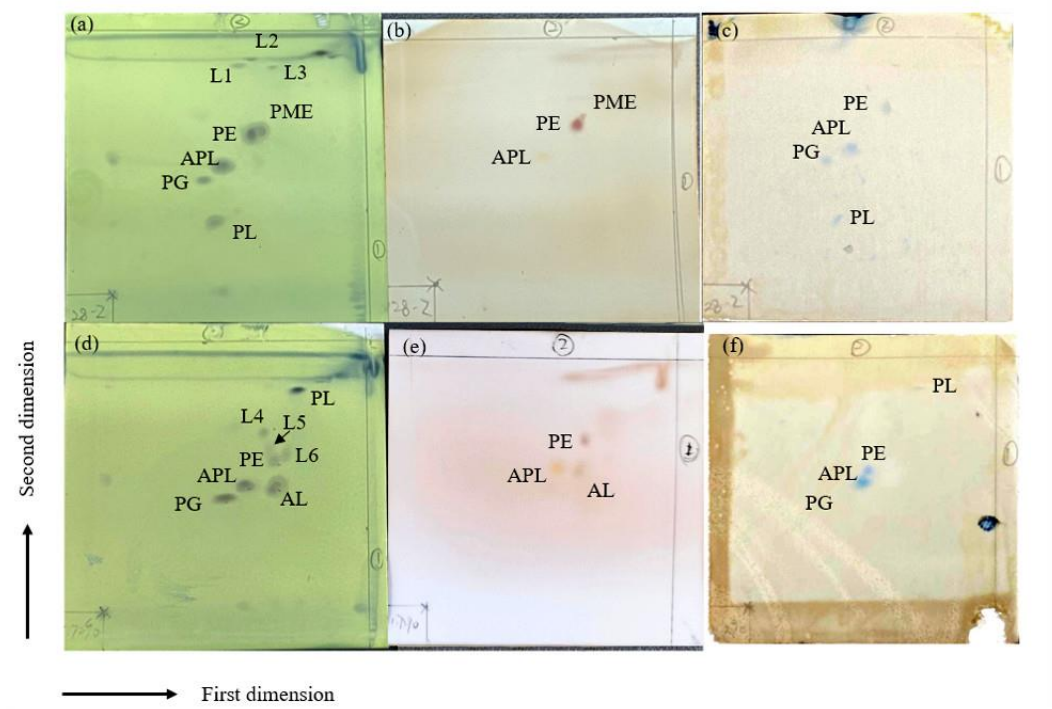

Supplementary Table S1. The poly-β-hydroxybutyrate synthesis related genes of the *Mameliella* strains

Strains: 1, *Mameliella* sp. DP3N28−2^T; 2, *M. alba* JLT354-W^T. Genes: *phaC*, polyhydroxyalkanoate synthase gene; *phaZ*, polyhydroxyalkanoate depolymerase gene; *phaR*, polyhydroxyalkanoate regulator gene; *phaP*, polyhydroxyalkanoate granule-associated phasin gene.

**Gene**	**1**	**2**
phaC	WP_218451276.1	WP_043138269.1
	WP_218451025.1	
phaZ	WP_218451275.1	WP_209037549.1
phaR	WP_218451861.1	WP_210426244.1
phaP	WP_218451277.1	WP_043138270.1
	WP_218451278.1	

Supplementary Table S2. The detailed results of API stripe tests and Biolog GEN III test for the *Mameliella* strains

Strains: 1, *Mameliella* sp. DP3N28−2^{T}; 2, *M. alba* CGMCC 1.7290^{T}. All data were obtained from this study unless otherwise indicated.

**Characteristics**	**1**	**2**
**API ZYM**		
Alkaline phosphatase	+	+
Esterase (C4)	+	+
Esterase lipase (C8)	+	w/+ [a]
Lipase (C14)	+	+
Leucine arylamidase	+	+
Valine arylamidase	+	+
Cystine arylamidase	+	+
Trypsin	−	−
α-Chymotrypsin	−	−
Acid phosphatase	+	+
Naphtol-AS-BI-phosphohydrolase	+	w/+ [a]
α-Galactosidase	+	−
β-Galactosidase	−	−
β-Glucuronidase	−	−
α-Glucosidase	−	−
β-Glucosidase	−	−
N-Acetyl-β-glucosaminidase	−	−
α-Mannosidase	−	−
α-Fucosidase	−	−
**API 20NE**		
Reduction of nitrate to nitrite	−	+
Denitrification	−	−/+ [a]
Indole production	−	−
D-Glucose fermentation	+	+/− [a]
Arginine dihydrolase	−	−
Urease	+	+
β-Glucosidase (Aesculin hydrolysis)	+	+
Gelatin hydrolysis	w	+
β-Galactosidase	+	w/+ [a]
D-Glucose	w	+
L-Arabinose	w	+
D-Mannose	w	−/+ [a]
D-Mannitol	−	+
N-Acetyl-glucosamine	−	−
D-Maltose	−	+
Potassium gluconate	−	+

Capric acid	−	−
Adipic acid	−	w/+
Malic acid	−	w/+
Trisodium citrate	−	−/+
Phenylacetic acid	−	+/+− [a]
**Biolog Gen III**		
Negative-control	–	–
D-raffinose	+	+
α-D-glucose	+	–
D-sorbitol	+	–
Gelatin	–	–
Pectin	+	–
p-hydroxy-phenylacectic acid	+	–
Tween 40	–	–
Dextrin	–	–
D-lactose	+	–
D-mannose	+	–
D-mannitol	+	–
Glycyl- L-proline	–	–
D-galacturonic acid	+	+
Methyl pyruvate	+	–
γ-Amino-butyric acid	+	–
D-Maltose	–	–
D-Melibiose	+	–
D-Fructose	+	–
D-Arabitol	+	–
L-Alanine	+	+
L-Galactonic acid lactone	+	+
D-Lactic acid methyl ester	–	–
α-Hydroxy-butyric acid	+	+
D-Trehalose	+	–
β-Methyl-d-glucose	+	–
D-Galactose	–	–
myo-Inositol	+	–
L-Arginine	+	–
D-Gluconic acid	+	–
L-Lactic acid	+	–
β-Hydroxy-DL-butyric acid	+	–
D-Cellobiose	+	–
D-Salicin	+	–
3-Methyl glucose	–	–
Glycerol	–	–
L-Aspartic acid	+	–
D-Glucuronic acid	+	+

Citric acid	–	–
α-Keto-butyric acid	+	–
Gentiobiose	+	–
N-Acetyl-D-glucosamine	–	–
D-Fucose	+	–
D-Glucose-6-PO4	+	–
L-Glutamic acid	+	–
Glucuronamide	+	+
α-Keto-glutaric acid	+	+
Acetoacetic acid	+	–
Sucrose	+	–
N-Acetyl-β-D-mannosamine	–	–
L-Fucose	+	–
D-Fructose-6-PO4	+	+
L-Histidine	–	–
Mucic acid	–	–
D-Malic acid	–	–
Propionic acid	+	–
D-Turanose	+	–
N-Acetyl-D-galactosamine	–	–
L-Rhamnose	–	–
D-aspartic acid	–	–
L-Pyroglutamic Acid	–	–
Quinic acid	–	–
L-Malic acid	+	+
Acetic acid	+	+
Stachyose	+	–
N-Acetyl neuraminic acid	–	+
Inosine	+	–
D-Serine	–	–
L-Serine	+	–
D-saccharic acid	–	+
Bromo-succinic acid	+	–
Formic acid	–	–
Positive-control	+	+
1% NaCl	+	+
1% Sodium lactate	+	+
Troleandomycin	–	–
Lincomycin	+	–
Vancomycin	+	–
Nalidixic acid	–	–
Aztreonam	–	–
PH6	+	–
4% NaCl	+	+

Fusidic acid	+	–
Rifamycin SV	+	–
Guanidine HCl	+	–
Tetrazolium-violet	+	–
Lithium chloride	+	+
Sodium butyrate	–	–
PH5	+	–
8% NaCl	+	–
d-serine	+	–
Minocycline	+	–
Niaproof 4	–	–
Tetrazolium blue	+	–
Potassium tellurite	–	+
Sodium bromate	+	+

[a], Data after dash are the results based on the five *M. alba* strains from reference [8].
